# Reflection and Transmission Coefficient of Yttrium Iron Garnet Filled Polyvinylidene Fluoride Composite Using Rectangular Waveguide at Microwave Frequencies

**DOI:** 10.3390/ijms13078540

**Published:** 2012-07-09

**Authors:** Hassan Soleimani, Zulkifly Abbas, Noorhana Yahya, Kamyar Shameli, Hojjatollah Soleimani, Parvaneh Shabanzadeh

**Affiliations:** 1 Fundamental and Applied Science Department, Universiti Teknologi PETRONAS, Seri Iskandar, 31750, Perak, Malaysia; E-Mail: Noorhana_yahya@petronas.com.my; 2 Department of Physics, Faculty of Science, Universiti Putra Malaysia, Serdang, 43400 UPM, Selangor, Malaysia; E-Mails: Za@science.upm.edu.my (Z.A.); hojjatsoleimani@yahoo.com (H.S.); 3 Department of Chemistry, Faculty of Science, Universiti Putra Malaysia, Serdang, 43400 UPM, Selangor, Malaysia; 4 Material & Energy Research Center, Alborz, Karaj, 3177983634, Iran; 5 Department of Chemical Engineering, Faculty of Engineering, Islamic Azad University, Malard Branch, 3169153174, Iran; E-Mail: parvaneh.shabanzade@gmail.com

**Keywords:** microwave measurements, RW-90 waveguide, YIG, PVDF, composites

## Abstract

The sol-gel method was carried out to synthesize nanosized Yttrium Iron Garnet (YIG). The nanomaterials with ferrite structure were heat-treated at different temperatures from 500 to 1000 °C. The phase identification, morphology and functional groups of the prepared samples were characterized by powder X-ray diffraction (PXRD), scanning electron microscopy (SEM) and Fourier transform infrared spectroscopy (FT-IR), respectively. The YIG ferrite nanopowder was composited with polyvinylidene fluoride (PVDF) by a solution casting method. The magnitudes of reflection and transmission coefficients of PVDF/YIG containing 6, 10 and 13% YIG, respectively, were measured using rectangular waveguide in conjunction with a microwave vector network analyzer (VNA) in X-band frequencies. The results indicate that the presence of YIG in polymer composites causes an increase in reflection coefficient and decrease in transmission coefficient of the polymer.

## 1. Introduction

Ferrite loaded polymer nanocomposites with permittivity less than 10 are increasingly used in microwave devices such as isolators, filters and circulators [1–3]. Yttrium iron garnet (Y_3_Fe_5_O_12_) is a soft ferrite material with a broad range of applications in electronic devices due to its efficient absorption of electromagnetic waves, low saturation flux density, low losses at high frequencies, low permeability, high resistivity and because it also has ability to magnetize and demagnetize easily. Therefore, much interest have been focused on polymer-based composites filled with ferrite particles, such as Co-ferrite [4], NiZn-ferrite [5], and MnZn-ferrite [6,7]. Various methods are used in the synthesis of nanoparticles; the sol-gel method was chosen in this research because of its low cost, simplicity, reproducibility and ability to control properties and structure by changing parameters such as stirring period, annealing temperature, precursor material, type of solvent and many more [8,9].

The transmission/reflection rectangular waveguide method (TR) is applied to obtain the reflection and transmission coefficients for the ferrite materials [10,11]. In this method, an isotropic material sample with specific length is positioned in rectangular waveguide. The reflection and transmission coefficients are defined by using an automatic network analyzer (VNA) in X-band frequencies [12–15].

This paper reports the results obtained by synthesis and characterization of nanostructured Yttrium Iron Garnet (YIG) using the sol-gel method. Moreover, the as-prepared material was composited with PVDF as a polymeric matrix due to its flexibility and easy processability to provide the carrier template. Reflection and transmission coefficients were also determined at various percentages of PVDF in YIG filled composites.

## 2. Results and Discussion

### 2.1. Powder X-ray Diffraction

**Figure 1:**
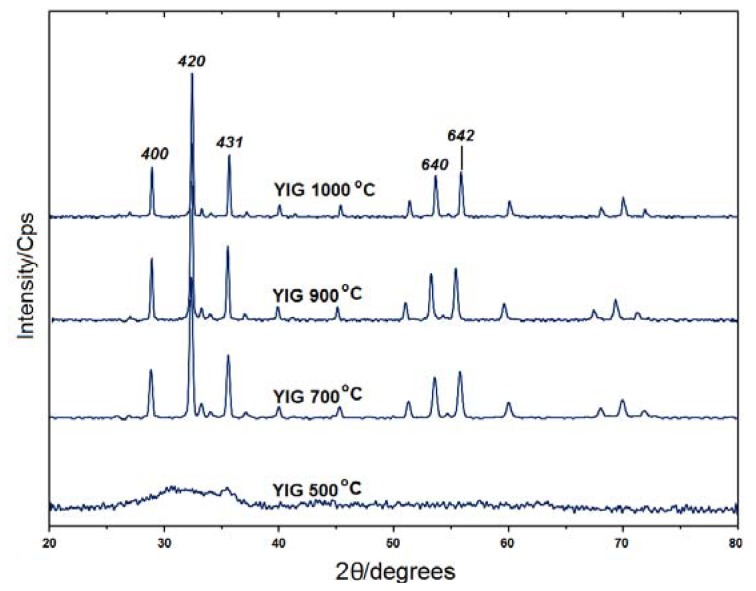
Powder X-ray diffraction (PXRD) patterns for the heat-treated products of the as-synthesized Yttrium Iron Garnet (YIG) at various temperatures.

X-ray Diffraction (XRD) patterns for the as-prepared YIG after the heat-treatment process at various temperatures are presented in Figure 1. The obtained product at 500 °C is an amorphous material due to the lack of peaks accompanied with YIG. The mean crystallite sizes of YIG at 700 °C, 900 °C and 1000 °C using 420 plane were estimated to be about 53.24, 53.35 and 66.56 nm, respectively, using Debye-Scherrer’s formula. The observed d-spacing values of the samples obtained at various temperatures for the 420 plane are listed in Table 1. These values are matched with the standard d-spacing values for the material (JCPDS No. 01-077-1998).

As the mean crystallite size decreases with increasing full width at half of the maximum intensity (FWHM), the values of FWHM are almost the same for YIG samples heated at 700 and 900 °C. This explains why the mean crystallite sizes for the sample heated at 1000 °C is larger than that of the samples heated at lower temperatures. This is attributed to crystallite growth at high temperature.

### 2.2. Surface Morphology Study

**Figure 2:**
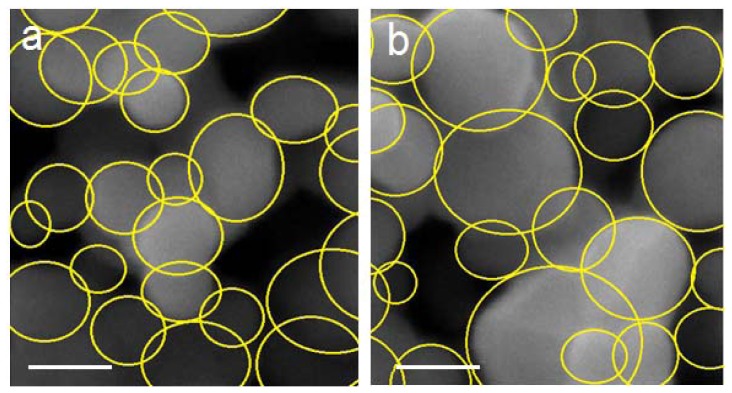
Scanning electron microscope (SEM) image of YIG at 700 and 1000 °C (**a**,**b**), the scale bar is 250 nm.

Figure 2a,b shows images of the heat-treated YIG at 700 and 1000 °C obtained from SEM. The figures shows nanosized particles with irregular shapes which confirm the results obtained by PXRD analysis.

### 2.3. Surface Chemistry (FT-IR)

**Figure 3:**
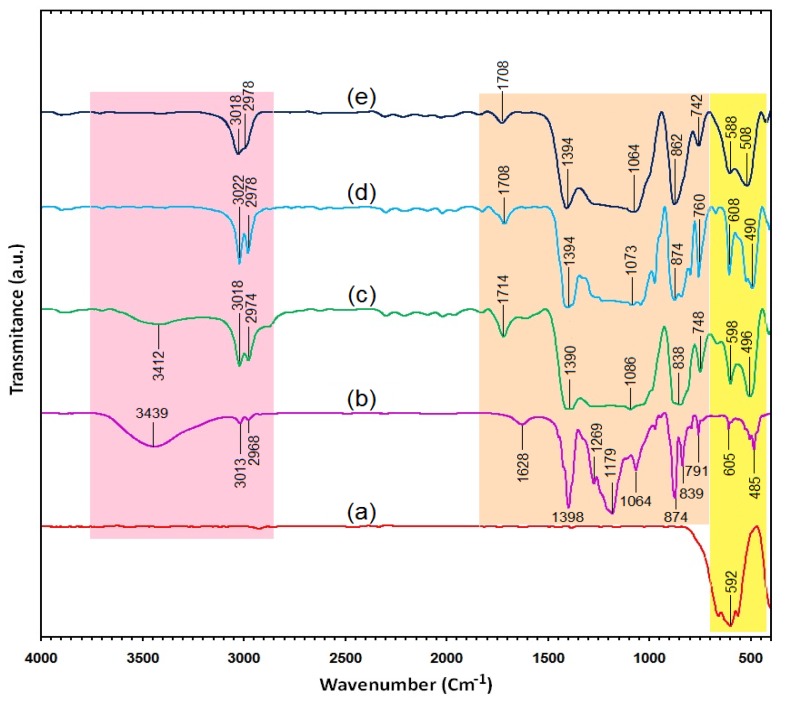
Fourier transforms infrared spectroscopy (FT-IR) spectra for YIG (**a**), PVDF (**b**) and PVDF/YIG containing 6, 10 and 13% YIG (**c**–**e**) composites respectively.

Figure 3a shows the FT-IR spectra for YIG, PVDF and PVDF/YIG containing 6, 10 and 13% YIG, respectively. The FT-IR spectrum of YIG shows sharp and narrow bands at 654, 592 and 558 cm^−1^, which can be attributed to the metal oxide, M-O stretching vibrations [16,17].

The spectrum of original PVDF, as depicted in Figure 3b, exhibited the following characteristic intense bands: 1398 cm^−1^, 1269 cm^−1^, 1179 cm^−1^ and 874 cm^−1^ for C-H out of plane deformation vibrations, 1065 cm^−1^ for C-C vibrations, 839 cm^−1^ and 791 cm^−1^ for CH_2_ rocking vibrations, 791 cm^−1^ for CF_2_ bending and skeletal vibrations [18]. The broad band at 3439 cm^−1^ is attributed to the O-H stretching vibration of the hydroxyl group of physically adsorbed water molecules [18,19]. Also, short bands at 3013 cm^−1^ and 2968 cm^−1^ relative to C-H stretching and 1464 and 1343 cm^−1^ for C-H bending vibrations.

Figure 3c–e also shows the FT-IR spectra of both PVDF/YIG (6, 10 and 13%) as the as-synthesized composites. As expected, the spectra are generally composed of combined spectral band features of YIG and PVDF. This indicates that both functional groups of YIG and PVDF are simultaneously present in both composites.

The O-H stretching vibrations with a small and broad peak that appeared at 3412 cm^−1^ for PVDF/YAG (6%) disappeared when the percentage was increased to 10 and 13%, respectively. This is due to a decrease in the O-H group interaction with YIG composites. In addition, the C-H stretching in the PVDF/YIG composites appeared in the range of 3018 cm^−1^ to 2968 cm^−1^ for asymmetric and symmetric vibrations. The absorption bands around 1390–1064 cm^−1^ are attributed to the C-F stretching vibration in the composites. The shifting of the bands, as compared to those of the free PVDF, reveals the presence of hydrogen bonding between the oxygen groups of YIG as a filler fluoride group in PVDF as a polymeric matrix. The bands at 838, 874 and 862 cm^−1^ for different percentages of PVDF/YIG show the C-H rock vibrations that shifted to the low wavenumber. The two bands around 508–608 cm^−1^ can be attributed to the M-O and the filler material and YIG. This shifting, when compared with the bare YIG, also reveals the presence of hydrogen bonding between YIG and PVDF. There are not many differences between the FT-IR bands for the two composites. This is owing to the inability of the FT-IR technique to provide a quantitative analysis [20].

### 2.4. Reflection and Transmission Coefficient

**Figure 4:**
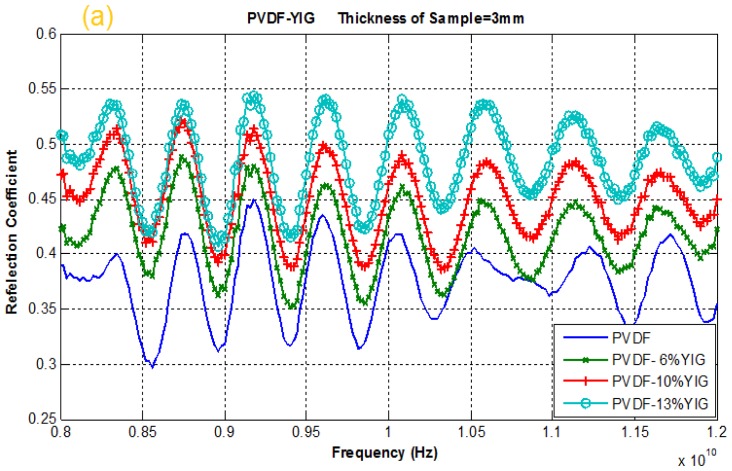

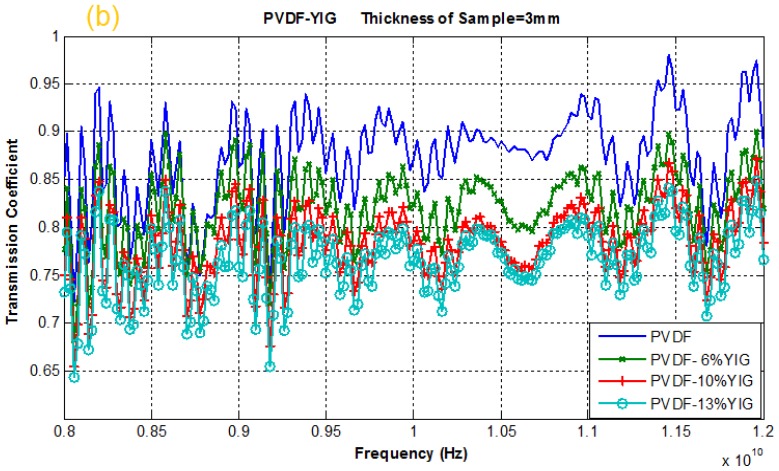
Variation of (**a**) reflection and (**b**) transmission coefficient of 3 mm thick PVDF-YIG composites (6, 10 and 13%) placed at rectangular waveguide at X-band frequencies.

The variations of reflection and transmission coefficient values with different frequencies for various percentage YIG filled PVDF-polymer composites placed at rectangular waveguide cross section are presented in Figure 4a,b. As shown in Figure 4, the *X*-axis represents the frequency from 8 to 12 GHz and *Y*-axis indicates a tolerance from zero to one. There are two main curves in these diagrams, namely transmission and reflection coefficient whose sum of curve values is almost around unity.

As expected, the reflection coefficient of PVDF was increased when it was mixed with YIG over the entire frequency range [14,15]. It can be deduced that an increased value of YIG in PVDF leads to an increase in the magnitude of reflection coefficient and a decreases in magnitude of transmission coefficient. Hence, samples with higher percentages of YIG resulted in higher reflection and lower transmission. For PVDF-13% YIG, the transmission coefficients are 0.65 and 0.84 in lowest and highest values of the mentioned frequencies, respectively, and the reflection coefficients are 0.39 and 0.59 in lowest and highest values of the frequencies, respectively. Meanwhile, PVDF-6% YIG demonstrates a transmission coefficient as high as 0.87 and reflection coefficient as low as 0.36. Consequently, the results indicate that the addition of YIG to polymer composite increase the reflection coefficient and decreases the transmission coefficient of the polymer [4,7]. It can be deduced that increasing the real and imaginary parts of permittivity and permeability of PVDF by adding the ceramic particles on PVDF-polymer composite at 8 to 12 GHz frequencies causes the reflection coefficient of YIG filled PVDF-polymer composite to increase while the transmission coefficient decreases [21].

## 3. Experimental Section

### 3.1. Materials and Method

YIG were prepared according to previous work [16]. Briefly, 13.89 g Fe(NO_3_)_3_·9H_2_O (Merck, 99%) and 8.92 g Y(NO_3_)_3_·6H_2_O (Merck, 96.0%) were dissolved in 38.86 g of citric acid and then distilled water was added to obtain 250 mL solution gel, the resulting solution was heated at 80 °C under stirring. The obtained gel was dried at 110 °C in an oven for 24 hours to remove water and trapped gases. Prior to the heat-treatment process, the sample was pre-heated at 100 °C lower than the heat-treatment temperatures. The pre-heated powders were wet crushed for 6 hours using a planetary micromill to obtain fine and homogenous particles. The dried sample was subjected to the heat-treatment temperatures of 500, 700, 900 and 1000 °C in air to form crystalline garnet phase (Vecstar Muffle Furnace). Polyvinylidene fluoride (PVDF) was used as polymeric matrix with dielectric constant of about 5 at 10 MHz to produce PVDF/YIG of 6, 10 and 13% respectively, as composites samples by solvent method.

### 3.2. Characterization

Powder X-ray diffraction patterns of the prepared materials were collected on a Philips, X′ Pert-MPD system equipped with a graphite monochromator, operating at 40 kv and 30 mA. A nickel-filtered CuKα radiation (λ = 0.1542 nm) was used. Microstructure analysis in this study was carried out to investigate the surface morphology and also to obtain the particle size of samples prepared. LEO 1455VP scanning electron microscope (SEM) was employed for this study. Chemical bonding information on various functional groups was investigated with Fourier transform infrared spectroscopy (Perkin-Elmer BX II FT-IR spectrophotometer in the range, 400–4000 cm^−1^) using the potassium bromide (KBr) pellet technique.

The composite samples mentioned earlier with dimensions of 22.86 × 11.43 and thickness of 3 mm, were snugly fit into a WR-90 waveguide, the reflection and transmission coefficients were measured in the frequency range of 8–12 GHz by using an Agilent N5230A PNA-L network analyzer. In this (T/R rectangular waveguide) method, the fundamental transverse electromagnetic (TEM) mode is the only mode that propagates in rectangular waveguide. Network analyzer was calibrated by implementing a standard full two-port calibration technique (SOLT) for 201 frequency points in X-band. All experiments were carried out at room temperature.

## 4. Conclusions

Yttrium iron garnet nanoparticles were successfully prepared by the sol-gel method. To obtain the crystalline phase, the material was heat-treated at 500–1000 °C. The high purity and crystallinity of the sol-gel derived YIG samples were confirmed by XRD and SEM techniques. The magnitudes of reflection and transmission coefficients of PVDF-YIG (6, 10 and 13% YIG) for a 3 mm thick sample were successfully measured at X-band frequencies using the (T/R) rectangular waveguide technique. From general observation of the results obtained from the analysis, one can conclude that transmission coefficients are generally higher than reflection coefficients. Furthermore, it is also conclusive that a composition of a higher percentage of YIG in the polymer results in a higher reflection coefficient and lower transmission coefficient responses.

## Figures and Tables

**Table 1:** Data from X-ray Diffraction (XRD) of Yttrium Iron Garnet (YIG) at various temperatures based on 420 plane (maximum peak)

Temperature	2θ	d-Spacing (A)	FWHM	Crystallite Size D (nm)^a^
500 °C	*	*	*	*
700 °C	32.3280	2.76930	0.1624	53.24
900 °C	32.3592	2.76670	0.1624	53.35
1000 °C	32.3441	2.76795	0.1299	66.56

a The calculations were conducted based on the 420 plane;

* Amorphous material; FWHM = full width at half of the maximum intensity.
